# Prediabetes as a risk factor for new-onset atrial fibrillation: the propensity-score matching cohort analyzed using the Cox regression model coupled with the random survival forest

**DOI:** 10.1186/s12933-023-01767-x

**Published:** 2023-02-20

**Authors:** Jung-Chi Hsu, Yen-Yun Yang, Shu-Lin Chuang, Lian-Yu Lin, Tony Hsiu-Hsi Chen

**Affiliations:** 1 Division of Cardiology, Department of Internal Medicine, Fu Jen Catholic University Hospital, Fu Jen Catholic University, New Taipei City, Taiwan; 2 Division of Cardiology, Department of Internal Medicine, National Taiwan University College of Medicine and Hospital, No.7, Chung-Chan South Road, Taipei, 100 Taiwan; 3 Department of Medical Research, National Taiwan University Hospital, Taipei, Taiwan; 4 Department of Internal Medicine, College of Medicine, National Taiwan University, Taipei, Taiwan; 5 Institute of Epidemiology and Preventive Medicine, College of Public Health, National Taiwan University, Taipei, Taiwan

**Keywords:** Prediabetes, Atrial fibrillation, Heart failure, Random survival forest

## Abstract

**Background:**

The glycemic continuum often indicates a gradual decline in insulin sensitivity leading to an increase in glucose levels. Although prediabetes is an established risk factor for both macrovascular and microvascular diseases, whether prediabetes is independently associated with the risk of developing atrial fibrillation (AF), particularly the occurrence time, has not been well studied using a high-quality research design in combination with statistical machine-learning algorithms.

**Methods:**

Using data available from electronic medical records collected from the National Taiwan University Hospital, a tertiary medical center in Taiwan, we conducted a retrospective cohort study consisting 174,835 adult patients between 2014 and 2019 to investigate the relationship between prediabetes and AF. To render patients with prediabetes as comparable to those with normal glucose test, a propensity-score matching design was used to select the matched pairs of two groups with a 1:1 ratio. The Kaplan–Meier method was used to compare the cumulative risk of AF between prediabetes and normal glucose test using log-rank test. The multivariable Cox regression model was employed to estimate adjusted hazard ratio (HR) for prediabetes versus normal glucose test by stratifying three levels of glycosylated hemoglobin (HbA1c). The machine-learning algorithm using the random survival forest (RSF) method was further used to identify the importance of clinical factors associated with AF in patients with prediabetes.

**Results:**

A sample of 14,309 pairs of patients with prediabetes and normal glucose test result were selected. The incidence of AF was 11.6 cases per 1000 person-years during a median follow-up period of 47.1 months. The Kaplan–Meier analysis revealed that the risk of AF was significantly higher in patients with prediabetes (log-rank p < 0.001). The multivariable Cox regression model indicated that prediabetes was independently associated with a significant increased risk of AF (HR 1.24, 95% confidence interval 1.11–1.39, p < 0.001), particularly for patients with HbA1c above 5.5%. The RSF method identified elevated N-terminal natriuretic peptide and altered left heart structure as the two most important risk factors for AF among patients with prediabetes.

**Conclusions:**

Our study found that prediabetes is independently associated with a higher risk of AF. Furthermore, alterations in left heart structure make a significant contribution to this elevated risk, and these structural changes may begin during the prediabetes stage.

**Supplementary Information:**

The online version contains supplementary material available at 10.1186/s12933-023-01767-x.

## Introduction

Atrial fibrillation (AF) has a significant impact on cardiovascular outcomes and is associated with high levels of morbidity and mortality [1, 2]. Studies have shown that individuals with diabetes mellitus (DM) are at an increased risk of developing AF. Metabolic abnormalities present in individuals with DM, such as insulin resistance, proinflammation, and abnormal hemostasis, can lead to endothelial dysfunction and atherogenesis, making them more susceptible to atrial fibrosis, autonomic dysfunction, and maladaptive myocardial remodeling [3–6]. Generally, individuals with DM have a 35% higher risk of developing AF [7].

The concept of "glycemic continuum" refers to the reciprocal relationship of a gradual decline in insulin sensitivity giving a rise in glucose levels [8]. The criteria for diagnosing prediabetes have changed over time and vary between organizations, such as the World Health Organization (WHO) or American Diabetes Association (ADA). Impaired fasting glucose (IFG) and impaired glucose tolerance (IGT) were the most commonly used criteria previously, and glycosylated hemoglobin (HbA1c) levels have been added as a new diagnostic criterion for prediabetes since 2010. It is estimated that 5–10% of the people with IFG/IGT-based prediabetes progress to DM annually, with 70% eventually developing DM at some point in their lifetime [9]. Although prediabetes on the glycemic continuum does not meet the criteria for DM, patients in this category are still susceptible to chronic inflammatory processes, leading to endothelial dysfunction and insulin resistance [10].

End-organ damage can occur even when HbA1c levels are below the threshold for DM. A meta-analysis found that prediabetes based on IFG or IGT was associated with a higher risk of cardiovascular disease [11]. Exposure to chronic hyperglycemia led to a two-fold increase in the risk of long-term mortality and cardiovascular events [12]. Prediabetes is associated with a gradient of risk for atherosclerotic cardiovascular disease, chronic kidney disease (CKD), and heart failure (HF) across different HbA1c levels [13, 14]. Individuals with prediabetes have been found to have alterations in cardiac structure and function, such as increased left ventricular mass and impaired diastolic function. Some high-risk patients may develop cardiovascular and kidney complications without progression to frank DM [15–17].

Previous studies have revealed that AF may be linearly correlated with serum HbA1c levels, and that patients with prediabetes have worse outcomes related to AF, such as ischemic stroke. Prediabetes with AF confers a higher risk of cerebrovascular events, even after adjusting for CHA2DS2-VASc risk factors [18, 19]. However, it remains unclear whether prediabetes is independently associated with AF, particularly with respect to the time of occurrence.

The first aim of this study was to investigate whether prediabetes, independent of other risk factors, increases the risk of developing AF over time using the traditional Cox regression model. The second aim was to identify significant clinical correlates responsible for the development of AF in prediabetes using a tree-based decision survival method, such as the random survival forest (RSF) machine-learning algorithm.

## Methods

### Study population

We conducted a longitudinal retrospective cohort study using the electronic health records (EHRs) of Taiwanese individuals aged 45 years or older who received care at a tertiary medical center between January 1, 2014 and December 31, 2019. The EHRs were obtained from the National Taiwan University Hospital Integrated Medical Database (NTUH-iMD) [20, 21], which collects data from the Taipei Main Hospital and branches in at least four counties in Taiwan. EHRs have been digitized and are available online since 2006 and include information on patients' demographics, diagnoses, medical orders, laboratory results, interventions, medications, and examinations. The quality of medical data has been shown to be consistent in providing real-world evidence [22]. The study was approved by the Institutional Review Board of the National Taiwan University Hospital.

Patients without serum glucose tests were excluded from the study. Additionally, we excluded individuals with a history of AF, atrial flutter, or valvular heart disease. AF and its occurrence time were identified using the International Classification of Diseases (ICD) codes from the EHRs or through standard 12-lead electrocardiograms. At least one electrophysiologist examined the electrocardiograms and confirmed the presence of AF or atrial flutter. The ICD-10 code I48 for AF and flutter is a medical classification advocated by the WHO. According to the 2022 ADA guidelines, prediabetes was defined as a fasting plasma glucose (FPG) level between 100 mg/dL (5.6 mmol/L) and 125 mg/dL (6.9 mmol/L), a 2-h plasma glucose (PG) level between 140 mg/dL (7.8 mmol/L) and 199 mg/dL (11.0 mmol/L) during a 75 g oral glucose tolerance test (OGTT), or an HbA1c level between 5.7% and 6.4% (39–47 mmol/mol) [23]. To focus on the population with prediabetes and normal glucose test (NGT) results, we excluded individuals who were diagnosed with DM by ICD code or who had used any antidiabetic medication during the study period. For the purpose of our study, individuals who were diagnosed with AF on the same day as their glucose test were also excluded.

We evaluated baseline characteristics, including age, sex, body mass index (BMI), history of hypertension (HTN), hyperlipidemia, gout, HF, coronary artery disease (CAD), CKD, chronic obstructive pulmonary disease (COPD), peripheral arterial occlusive disease (PAOD), and transient ischemic attack (TIA)/ischemic stroke using data from the EHRs. The index date was defined as the date of the first recorded glucose test, including the FPG, 2-h PG, and HbA1c levels. Underlying conditions were defined as those diagnosed prior to the index date, using the ICD codes listed in the Additional file 1: Tables S1–S4. We also collected data on liver function, including alanine aminotransferase, renal function, lipid profile (total cholesterol [TCHO], low-density lipoprotein [LDL], high-density lipoprotein, triglyceride [TG]), and N-terminal-pro-B type natriuretic peptide (NT-pro-BNP), on or after the index date. The estimated glomerular filtration rate (eGFR) was calculated using the Modification of Diet in Renal Disease equation.

Echocardiographic studies were performed using the Phillips iE33 machine (Phillips, Bothell, WA, USA) and two-dimensional, M-mode measurements with a 3.0 or 3.5 MHz transducer. We collected measurements of the left atrium (LA) size, left ventricular internal dimension in end-diastole and systole, and left ventricular ejection function (LVEF) from the parasternal long-axis view using the M-mode cursor. LA size was measured as the anterior–posterior diameter at the end of ventricular systole. Left ventricular mass (LVM) was calculated using the Devereux’s formula. All echocardiographic data were obtained from the EHRs. The median follow-up period for echocardiographic data after the index date was 30.1 (13.0–49.5) and 28.4 (11.9–48.8) months in the NGT group and prediabetes group, respectively.

The outcome of this study was the first occurrence of AF after the index date. Mortality was adjudicated by a central committee. We reviewed all available medical records until the final clinical visit or death.

### Statistical analysis

The propensity-score matching (PSM) was used to render the two groups, NGT and prediabetes, as comparable as possible. Individuals with more than 70% of missing values were removed. Before PSM, we used the iterative nonparametric imputation method (MissForest) based on the random-forest algorithm [24] to impute large-scale mixed-type datasets. The propensity scores were estimated using logistic regression with optimal width [25]. We compared the covariate balance with that of the random forest method. A standardized mean difference of less than 10% was considered to be well matched.

Continuous variables were reported as mean (standard deviation), and categorical variables were reported as percentages. Differences among the groups were compared using chi-square tests for categorical variables and one-way analysis of variance tests for continuous variables. We also calculated the incidence, age-standardized incidence, incidence rate, and incidence rate ratio (IRR) of AF. The proportional hazards assumption was verified using the scaled Schoenfeld residuals and hazard ratio plots. We also examined possible nonlinear dose–response associations using restrictive cubic spline models in the Cox proportional hazards model. Multivariable Cox regression models were used to estimate hazard ratios (HRs) and 95% confidence intervals (CIs). The validity of the Cox proportional hazards regression model was confirmed using machine-learning-based imputations [26]. We adjusted for confounders in a stepwise manner to ensure consistency in the association through increasingly complex models. Model 1 was the crude model, and model 2 was further adjusted for baseline characteristics, including age, sex (with women as the reference group), BMI, HTN, hyperlipidemia, gout, CAD, COPD, PAOD, and prior history of TIA/ischemic stroke. In model 3, we further extended model 2 to include serum NT-pro-BNP levels and three echocardiogram parameters, including baseline LA size, LVEF, and LVM. Forest plots with adjusted HRs, CIs, and p-values were used for subgroup analyses. The estimated cumulative incidence of AF was derived using the Kaplan–Meier approach, and the significance of the difference between the curves was examined using log-rank tests.

The RSF method was used to construct a series of independent decision trees. Each tree was generated by randomly selecting a sample of patients from the original cohort and a subset of the variables. Log-rank statistics were used as the criteria for branching in all forests. The most important variables were determined by identifying those that frequently split branches near tree trunks. We evaluated nonparametric estimates and examined variable importance (VIMP) and minimal depth. The performance of predictions from each individual tree was measured using the Harrell's concordance index (C-index) using out-of-bag (OOB) data. We achieved stable and consistent results for the C-index after multiple iterations [27, 28].

To ensure that the result obtained from the RSF method was not inferior to other machine-learning methods with the classification of AF into two classes rather than time to AF, a series of machine-learning methods were also performed. These include the logistic regression model, decision tree method, support vector machine (SVM) with various kernels, and artificial neural network (ANN). All statistical analyses were conducted using R (version 4.1.2; University of Auckland, Auckland, New Zealand), SAS version 9.4 (SAS Institute Inc., Cary, NC, USA), and SPSS version 25.0 (SPSS Inc., Chicago, IL, USA). A p-value of less than 0.05 was considered statistically significant.

## Results

### Baseline characteristics

**Fig. 1:**
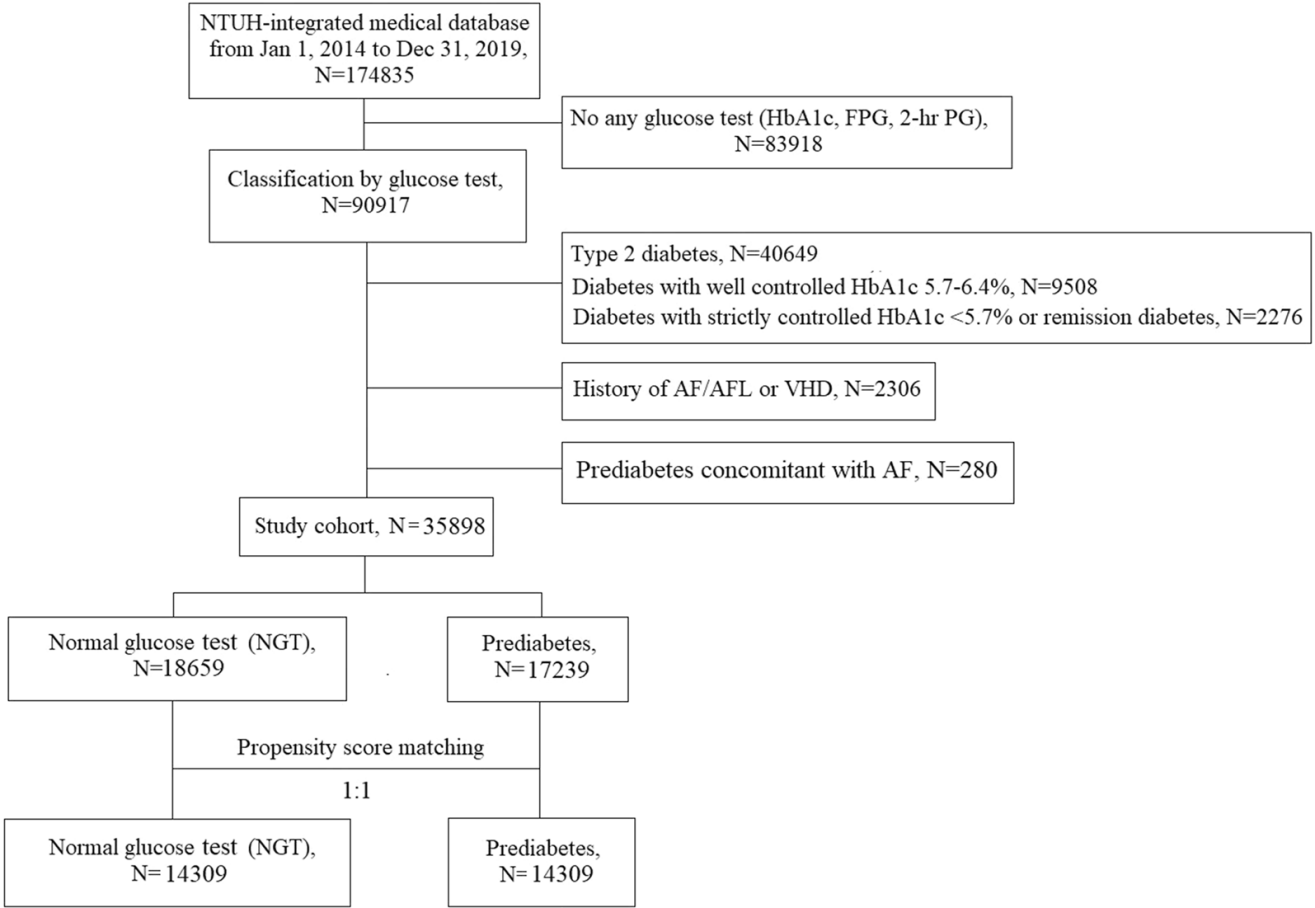
Study Flowchart

The process of patient selection for this study is illustrated in Fig. 1. A total of 174,835 individuals from the National Taiwan University Hospital were enrolled between 2014 and 2019. After excluding 83,918 patients without any glucose test, 52,433 patients who had a definite diagnosis of diabetes by ICD code or had received antidiabetic medications, and 2586 patients with preexisting or concurrent AF or valvular heart disease, 35,898 patients were eligible for analysis. Using the 2022 ADA guidelines, 17,239 individuals were classified into the prediabetes group, and 18,659 individuals were classified into the NGT group. After 1:1 PSM, each group included 14,309 participants.

The clinical, biochemical, and anthropometric characteristics of the participants are presented in Table 1. Before PSM, patients with prediabetes were older (65.0 ± 10.1 vs. 62.8 ± 10.3, p < 0.001), more likely to be men (49.1% vs. 40.9%, p < 0.001), had higher BMI (25.1 ± 3.8 vs. 23.7 ± 3.5, p < 0.001), and had more comorbidities, such as HTN (27.8% vs. 21.3%, p < 0.001), hyperlipidemia (19.7% vs. 14.9%, p < 0.001), CAD (12.6% vs. 8.6%, p < 0.001), and COPD (3.5% vs. 2.9%, p = 0.002). In contrast, patients with NGT had higher serum TCHO (189.3 ± 36.9 vs. 186.4 ± 38.2, p < 0.001) and LDL levels (113.5 ± 28.1 vs. 112.8 ± 29.7, p = 0.015) but had lower TG levels (120.8 ± 74.5 vs. 141.2 ± 87.2, p < 0.001). Patients with prediabetes had lower levels of NT-pro-BNP (1,134.9 ± 3106.4 vs. 1,542.4 ± 4120.1, p < 0.001) but a lower mean LVEF (51.4 ± 3.8 vs. 51.8 ± 5.4, p < 0.001) and higher mean LVM (210.4 ± 26.3 vs. 200.9 ± 23.7, p < 0.001). They also had a larger LA size (3.7 ± 0.3 cm vs. 3.6 ± 0.3 cm, p < 0.001) and higher FPG (105.9 ± 9.2 mg/dL vs. 90.0 ± 6.9 mg/dL, p < 0.001) and HbA1c levels (5.9 ± 0.2% vs. 5.4 ± 0.2%, p < 0.001) than the NGT group. After adjusting for confounders using PSM, the standard mean differences in variables between the groups were within 10%. Additional file 1: Figures S1A and B compare the covariate balance using standardized mean difference and show the results of PSM compared to logistic regression and random forest methods.

**Table 1:** Patients^,^ characteristics before and after propensity-score matching

	Pre-match				Post-match			
	NGT N = 18,659	Prediabetes N = 17,239	Standardized mean difference	p-value	NGT N = 14,309	Prediabetes N = 14,309	Standardized mean difference	p-value
Age (yr)	62.8 ± 10.3	65.0 ± 10.1	0.218	< 0.001	64.3 ± 10.5	64.6 ± 9.9	0.022	< 0.001
Sex (male)	7639 (40.9)	8463 (49.1)	0.163	< 0.001	6639 (46.4)	6770 (47.3)	0.018	0.121
BMI (kg/m^2^)	23.7 ± 3.5	25.1 ± 3.8	0.363	< 0.001	24.5 ± 3.6	24.8 ± 3.8	0.073	< 0.001
HTN	3967 (21.3)	4788 (27.8)	0.145	< 0.001	3505 (24.5)	3716 (26.0)	0.033	0.004
Hyperlipidemia	2771 (14.9)	3388 (19.7)	0.121	< 0.001	2430 (17.0)	2679 (18.7)	0.044	< 0.001
CAD	1602 (8.6)	2178 (12.6)	0.122	< 0.001	1448 (10.1)	1691 (11.8)	0.051	< 0.001
Gout	196 (1.1)	231 (1.3)	0.025	0.013	160 (1.1)	169 (1.2)	0.006	0.618
COPD	543 (2.9)	600 (3.5)	0.031	0.002	457 (3.2)	479 (3.3)	0.008	0.465
CKD	349 (1.9)	293 (1.7)	-0.013	0.232	254 (1.8)	257 (1.8)	0.002	0.893
PAOD	375 (2.0)	427 (2.5)	0.030	0.003	316 (2.2)	346 (2.4)	0.014	0.238
History of HF	56 (0.3%)	57 (0.3%)	0.005	0.638	41 (0.3%)	53 (0.4%)	0.015	0.215
History of TIA/ischemic stroke	234 (1.3)	283 (1.6)	0.031	0.002	200 (1.4)	213 (1.5)	0.007	0.519
TCHO (mg/dL)	189.3 ± 36.9	186.4 ± 38.2	-0.070	< 0.001	188.1 ± 37.3	187.6 ± 38.3	0.001	0.030
LDL (mg/dL)	113.5 ± 28.1	112.8 ± 29.7	0.008	0.015	113.7 ± 28.5	113.3 ± 28.5	-0.015	0.004
HDL (mg/dL)	53.9 ± 12.5	49.5 ± 11.8	-0.446	< 0.001	51.0 ± 10.8	50.4 ± 11.6	-0.053	< 0.001
TG (mg/dL)	120.8 ± 74.5	141.2 ± 87.2	0.264	< 0.001	130.9 ± 81.2	138.1 ± 86.2	0.082	< 0.001
ALT (U/L)	22.9 ± 31.9	25.8 ± 27.4	0.105	< 0.001	24.3 ± 35.7	25.1 ± 25.9	0.031	0.663
Baseline eGFR (mL/min/1.73 m^2^)	76.2 ± 24.9	75.8 ± 26.3	-0.012	0.137	75.9 ± 25.2	75.7 ± 25.9	-0.005	0.451
NT-pro-BNP (pg/mL)	1542.4 ± 4120.1	1134.9 ± 3106.4	-0.050	< 0.001	972.4.6 ± 3436.8	999.8 ± 3496.7	0.008	0.534
LA (cm)	3.6 ± 0.3	3.7 ± 0.3	0.330	< 0.001	3.6 ± 0.3	3.6 ± 0.3	0.078	< 0.001
LVEF (%)	51.8 ± 5.4	51.4 ± 3.8	-0.243	< 0.001	69.5 ± 4.8	68.9 ± 5.0	-0.122	< 0.001
LV mass (g)	200.9 ± 23.7	210.4 ± 26.3	0.430	< 0.001	177.3 ± 30.6	181.7 ± 32.2	0.136	0.006
Mean FPG (mg/dL)	90.0 ± 6.9	105.9 ± 9.2		< 0.001	90.4 ± 6.9	105.7 ± 9.0		< 0.001
Mean HbA1c (%)	5.4 ± 0.2	5.9 ± 0.2		< 0.001	5.4 ± 0.2	5.9 ± 0.2		< 0.001
AF/AFL	642 (3.4)	803 (4.7)		< 0.001	535 (3.7)	654 (4.6)		< 0.001

*NGT* normal glucose test, *BMI* body mass index, *HTN* hypertension, *CAD* coronary artery disease, *COPD* chronic obstructive pulmonary disease, *PAOD* peripheral artery disease, *CKD* chronic kidney disease, *PAOD* peripheral arterial occlusive disease, *TCHO* total cholesterol, *LDL* low-density lipoprotein, *HDL* high-density lipoprotein, *TG* triglyceride, *ALT* alanine aminotransferase, *eGFR* estimated glomerular filtration rate, *HF* heart failure, *TIA* transient ischemic accident, *NT-pro-BNP* N-terminal pro B type natriuretic peptide, *LVEF* left ventricular ejection fraction, *LA* left atrial size, *LV mass* left ventricle mass, *AF* atrial fibrillation, *AFL* atrial flutter

### Outcomes

Over a median follow-up period of 47.1 months, the overall cumulative incidence in the study population was 4.15%, with an incidence rate of 11.60%. The incidence of AF was 10.32 per 1000 person-years in the NGT group and 12.92 per 1000 person-years in the prediabetes group. The IRR was 1.25 (95% CI 1.12–1.40). After adjusting for age, the age-standardized incidences of AF in the NGT and prediabetes groups were 3.74% and 4.57%, respectively. Cumulative incidence and incidence rates are listed in Table 2.

**Table 2:** Cumulative incidence and incidence rates for atrial fibrillation (matched-cohort)

	N	Event	Incidence (%)	Age standardized incidence (%)	At risk (person-year)	Median follow-up time (month)	Incidence rate (event/1000 person-year) (95% CI)	Incidence rate ratio (IRR) (95% CI)
Overall population	28,618	1189	4.15	4.69	102,477	47.13	11.60 (10.95–12.28)	-
NGT	14,309	535	3.74	3.97	51,848	43.48	10.32 (9.46–11.23)	-
Prediabetes	14,309	654	4.57	5.46	50,629	42.46	12.92 (11.95–13.95)	1.25 (1.12–1.40)

*NGT* normal glucose test

**Fig. 2:**
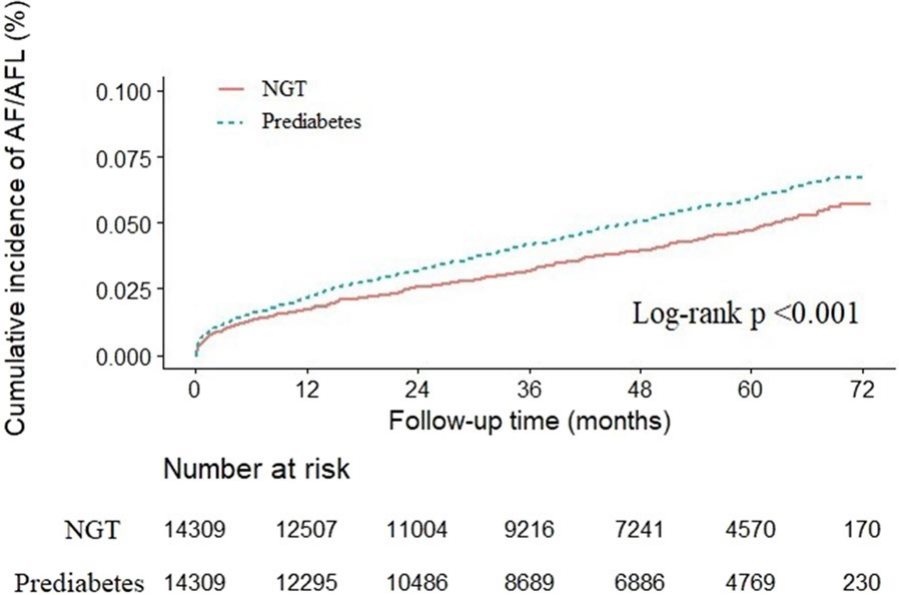
The Kaplan–Meier curve for the normal glucose test and prediabetes groups

Table 3 illustrates the risk of AF in the prediabetes group stratified by HbA1c level, using the NGT group as a reference. The prediabetes group had a significantly increased risk of AF by 24% (HR 1.24, 95% CI 1.11–1.39, p < 0.001) in the crude model. This elevated risk remained significant after adjusting for confounding factors in the fully adjusted model. Specifically, the risk of AF remained significant in individuals with prediabetes with higher HbA1c levels (HR 1.20, 95% CI 1.05–1.37, p = 0.007 and HR 1.36, 95% CI 1.17–1.59, p < 0.001 for HbA1c levels between 5.5–6.0% and 6.0–6.5%, respectively) but was not significant in the subgroup with HbA1c levels below 5.5% (HR 1.00, 95% CI 0.59–1.69, p = 0.996). The results of the Kaplan–Meier analysis, presented in Fig. 2, also support these findings, showing a significantly higher cumulative incidence of AF in patients with prediabetes (log-rank p < 0.001).

**Table 3:** Multivariable Cox regression for incidence of atrial fibrillation

	Prediabetes							
	Overall N = 28,618		HbA1c < 5.5% N = 315		HbA1c 5.5–6.0% N = 8,516		HbA1c 6.0–6.5% N = 5,478	
	HR (95% CI)	p-value	HR (95% CI)	p-value	HR (95% CI)	p-value	HR (95% CI)	p-value
Model 1	1.24 (1.11–1.39)	< 0.001	1.49 (0.89–2.48)	0.129	1.29 (1.13–1.47)	< 0.001	1.17 (1.01–1.36)	0.043
Model 2	1.25 (1.12–1.40)	< 0.001	1.38 (0.82–2.30)	0.222	1.30 (1.15–1.49)	< 0.001	1.16 (0.99–1.34)	0.061
Model 3	1.24 (1.11–1.39)	< 0.001	1.00 (0.59–1.69)	0.996	1.20 (1.05–1.37)	0.007	1.36 (1.17–1.59)	< 0.001

Reference group: NGT, normal glucose test (N = 14,309)

Model 1: crude

Model 2: adjusted for age, sex (ref: female), BMI, HTN, hyperlipidemia, gout, CAD, COPD, PAOD, eGFR, history of heart failure, and history of TIA/ischemic stroke

Model 3: Model 2 with further adjustment for NT-pro-BNP, LVEF, LA size, and LV mass

*HR* hazard ratio, *BMI* body mass index, *HTN* hypertension, *CAD* coronary artery disease, *COPD* chronic obstructive pulmonary disease, *PAD* peripheral artery disease, *eGFR* estimated glomerular filtration rate, *TIA* transient ischemic accident, *NT-pro-BNP* N terminal pro B type natriuretic peptide, *LVEF* left ventricular ejection fraction, *LA* left atrial size, *LV mass* left ventricle mass

**Fig. 3:**
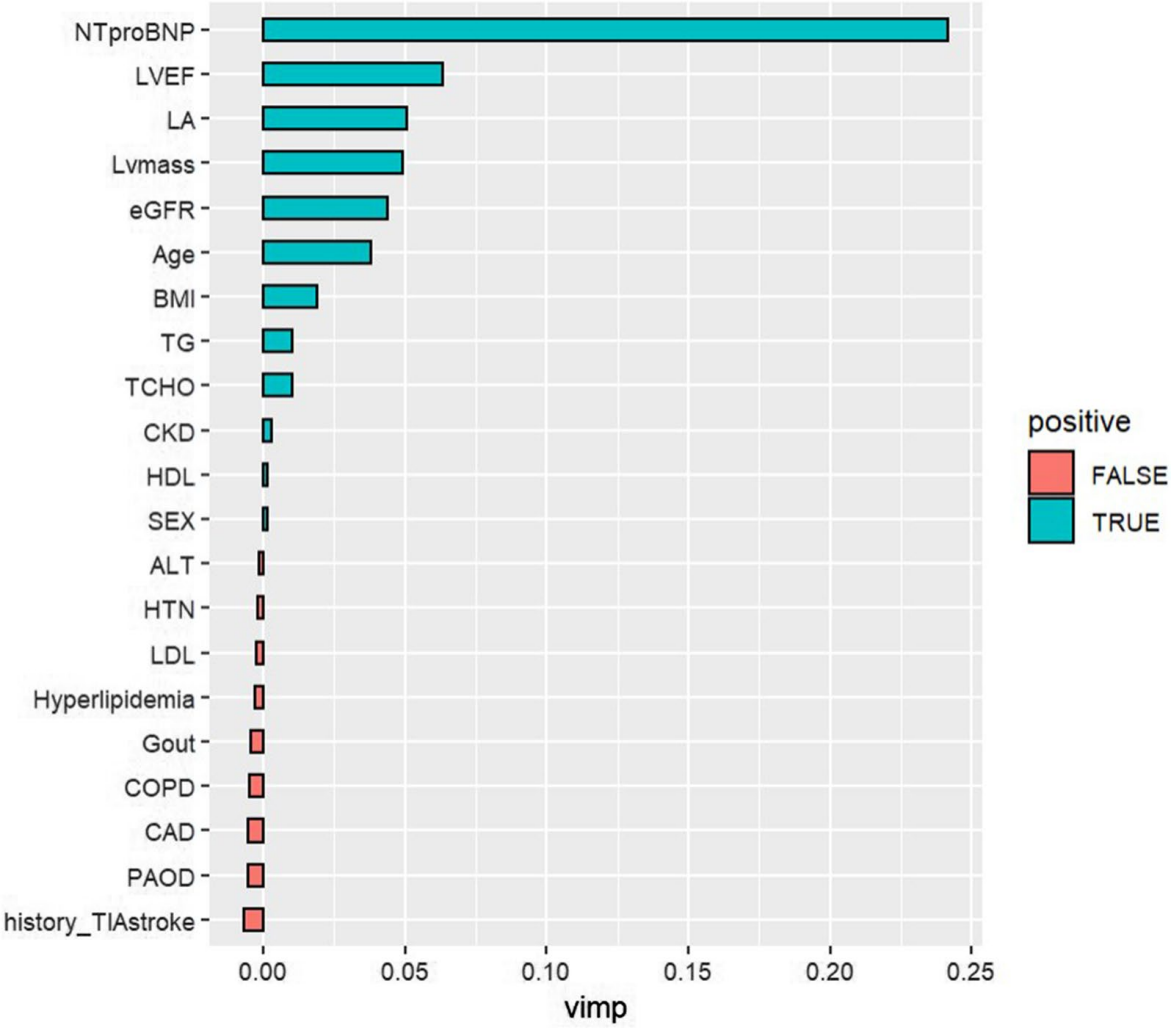
Variable importance for atrial fibrillation in patients with prediabetes

**Fig. 4:**
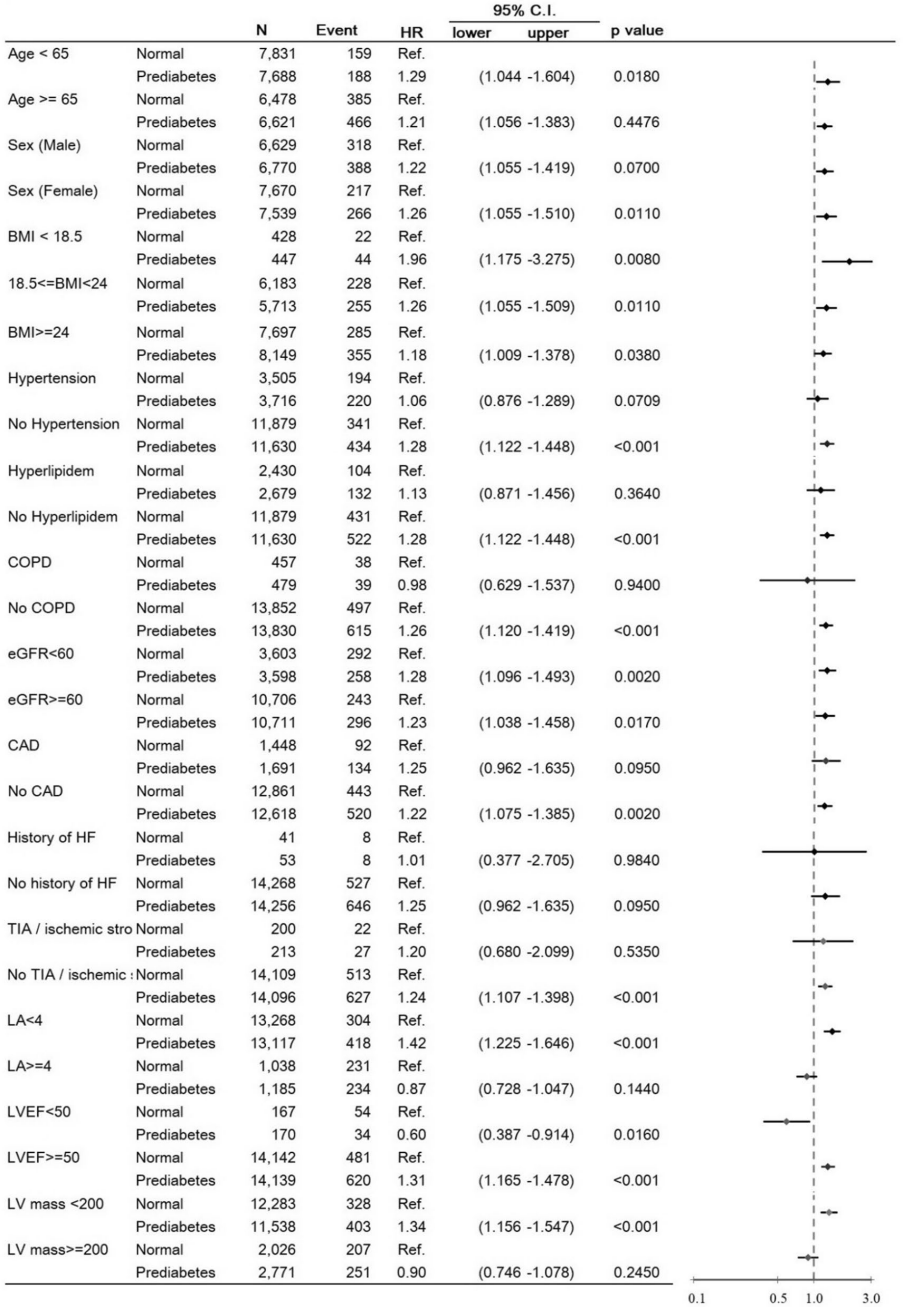
Subgroup analysis of the multivariable cox regression

The RSF planted 3,000 trees, and the average number of terminal nodes per tree was 432.2, with 21 variables used. The C-index for the corresponding area under curve (AUC) was 0.94. Fig. 3 illustrates VIMP. The factors positively associated with the development of AF were serum NT-pro-BNP levels, LVEF, LA size, LVM, eGFR, BMI, TG, and TCHO. The factors inversely associated with AF were HTN, LDL, hyperlipidemia, gout, COPD, CAD, PAOD, and a history of TIA/ischemic stroke. Fig. 4 presents a forest plot of the HRs derived from the Cox model for subgroup analyses.

As mentioned earlier in the statistical section, a series of machine-learning methods, in addition to the RSF method, were also performed. The results of model performance with the C-index were 0.80 for the logistic regression model, 0.84 for the decision tree, 0.86 for SVM with polynomial kernel, and 0.90 for ANN. It should be noted that different kernels of SVM showed different results. However, polynomial SVM gave the highest value of AUC. Regarding the identification of important variables, we selected the comparison between the decision tree model and RSF method as both have the same spirit of statistical machine learning. The most two important variables identified from the decision model were NT-pro-BNP and LA size, which are similar to the key factors identified from the RSF. The comparison of 0.94 obtained from the RSF with these corresponding figures using other machine-learning algorithms and the consistency with the identification of important variables using the decision tree method may suggest the adequacy of using the RSF method on this occasion.

## Discussion

Our study shows that individuals with prediabetes have an increased risk of developing AF, and that structural changes in the heart may progress along with the rise in glucose levels. These conclusions were reinforced by our use of a PSM design based on a retrospective cohort along with statistical models incorporating machine-learning methods.

Current definitions of prediabetes differ between the United States (ADA) and Europe (WHO). By using different criteria, previous studies have suggested that prediabetes is associated with an increased risk of cardiovascular disease or coexisting HF. The definition of prediabetes has changed over time, and diagnostic criteria vary among different guidelines. The ADA guidelines first applied an HbA1c of 5.7–6.4% as a new category for a higher risk for diabetes in 2010 [29]. However, it is still unclear which criteria are the best definitions of prediabetes related to cardiovascular outcomes. Some studies have shown that prediabetes defined by WHO-FPG is associated with a higher risk of cardiovascular disease, while others have found that IGT-defined prediabetes has an advantage in identifying high risk for all-cause mortality [30, 31]. IFG-defined prediabetes has been linked to the development of AF and coexisting HF [32, 33]. However, the Whitehall II study, a longitudinal follow-up study, found that more than 40% of OGTT-diagnosed diabetic patients did not meet the HbA1c criteria for DM but had similar risks of macro- and microvascular diseases compared to that of the diabetes-free population [34]. Currently, no study has investigated whether HbA1c-based diagnosis of prediabetes predict cardiovascular disease more accurately. This raises the question of whether HbA1c-defined prediabetes can accurately predict AF. In our study, we found that approximately one-third of the prediabetes cases were diagnosed using HbA1c levels. We found that HbA1c-defined prediabetes was associated with a higher incidence of AF. In the subgroup analysis, we also found that prediabetes with an HbA1c level below 5.5% had a nonsignificant association with AF. Even though the population below 5.5% was smaller, our finding may imply that prediabetes with low HbA1c levels may carry a lower risk of AF, and the cut-off point of HbA1c for this association may be approximately 5.5%.

Obesity is a significant contributor to cardiovascular diseases. Studies have shown that being overweight and having diabetes work together to provoke oxidative stress and harmful atrial remodeling, thereby increasing the risk of new-onset AF [35, 36]. It is well known that obesity increases the risk of AF; therefore, in patients with prediabetes, particularly those with multiple comorbidities, the risk of AF is expected to be even higher. However, in Asian populations, some studies have shown that the risk of new-onset AF is higher, particularly in patients with a BMI below 25 kg/m^2^ [32, 33]. Compared to individuals with classical obesity, subjects with "lean diabetes" have several distinct characteristics. For example, "lean (pre)diabetes" patients tend to have a rapid beta-cell failure rather than insulin resistance. They also have a high prevalence of "sarcopenic obesity," which is characterized by high body fat with reduced or normal body mass [37]. In this study, patients diagnosed with prediabetes had relatively low BMIs (24–25 kg/m^2^). We also observed that the risk of AF increased in patients with a BMIs lower than 25 kg/m^2^. These observations are consistent with previous Asian studies and emphasize the importance of recognizing prediabetes and its potential negative effects on cardiac structural remodeling.

Our findings suggest that left ventricular remodeling may occur earlier in the diabetes spectrum. Similar to our findings, a recent report using principle component analysis to analyze the high-dimensional characteristics of different stages of diabetes found that LVEF and left ventricular inner diameter were significantly changed at prediabetes stages compared to those with NGT [38]. Prediabetes has been linked to increased LVM, worse diastolic function, and subtle reductions in left ventricular systolic function [16]. Some biological mechanisms in prediabetes may contribute to the development of AF. First, metabolic disorders, such as diabetes, can simultaneously cause atrial and ventricular myopathy through cardiac inflammation and fibrosis [39]. Both AF and HF share common risk factors and comorbidities and are considered inflammatory diseases that may be exacerbated by hyperglycemia [40]. Second, neurohormonal imbalances, such as activation of the renin–angiotensin–aldosterone system and disturbances in electrophysiology due to changes in intracellular calcium handling in cardiac myocytes, can increase left atrial stretch and fibrosis and contribute to the development of AF [41].

As AF is a highly heterogeneous disease, identifying high-risk prediabetes and preventing its development are of great importance. Our findings may increase awareness among physicians regarding the care of patients with prediabetes. Screening and appropriate management of prediabetes may aid in the primary and secondary prevention of AF and its related complications.

The merits of methodology used in this study are three-fold. The first was to make use of propensity matching score design to render the two groups (prediabetes versus NGT) as comparable as possible in order to mimic a randomized controlled trial. The second was to use the traditional Cox regression model coupled with the RSF method, one of decision tree-based model that is widely used in statistical machining learning, especially in capturing complex and nonlinear relationships between the longitudinal and baseline variables regarding the hazard of the survival outcome [42]. The PSM design enhanced the validity of study results on the verification of prediabetes as an independent risk factor for AF. Using the tradition Cox regression model demonstrated that prediabetes independently increased the risk of AF. We then ranked the importance of several recognized risk predictors associated with AF over time by using the RSF method. The first was pertaining to the elevated NT-pro-BNP followed by altered left heart structure, including LVEF, LA size, and LA. The third was to consider a series of statistical machine-learning methods with emphasis of the classification of binary outcome or time to AF. Although each machine-learning method has its own merit, we still prefer the RSF method to other methods partly because of the highest value of AUC and partly because of our interest in first validating whether prediabetes is an independent risk factor associated with time to occurrence of AF rather than the binary outcome of whether to have AF only and then identifying key factors among patients with prediabetes. Such a thought prompted us to use the Cox regression model coupled with the RSF method for achieving the goal as mentioned above. The reason for the high AUC value in the RSF method may be that both the Cox regression model and RSF method are two methods tailored for dealing with time to AF in contrast to other methods, such as logistic regression decision tree, ANN, and SVM, which were used for the classification of AF. To consider time to AF for other methods, the deep machine-learning methods, such as recurrent neural network, can be considered in the future.

## Limitations

This study had some limitations. First, as it was a retrospective cohort study, there might have been surveillance bias, as the protocol for AF screening was not standardized. This might have led to underestimation of the incidence of new-onset AF. Second, this study did not evaluate certain known AF risk factors, such as sleep apnea and sedentary behavior. Having information on variables, such as smoking status, alcohol consumption, and physical activity level, may help improve the prediction of AF in the models. Finally, as this was a single-center study, it is unclear whether the findings can be generalized to a community setting.

## Conclusion

Our study revealed that individuals with prediabetes are at an increased risk of AF. Our results also indicate that changes in left heart structure are a significant contributor to this heightened risk and may commence during the prediabetes stage.

## Supplementary Information

**Additional file 1: Figure S1.** propensity score matching with logistic regression (A) and with random forest (B). **Figure S2.** Concordance between minimal depth and variable of importance (VIMP) for AF. **Figure S3.** Restrictive cubic spline for hazard ratio between AF and BMI in all patients (3A), model 1 crude for prediabetes (3B), model 2 for prediabetes (3C), model 3 full adjustment for prediabetes (3D). **Table S1.** Incidence of AF in transition diabetes status (the unmatched cohort). **Table S2.** C-index between traditional Cox model and random survival forest. **Table S3.** ICD-code for diagnoses. **Table S4.** ATC-codes for medications.

## Data Availability

The datasets used in this study are available only at the National Taiwan University Hospital. The R programs (codes) used in this study are available from the corresponding author upon request.

## References

[1] Schnabel RB, Yin X, Gona P, Larson MG, Beiser AS, McManus DD (2015). 50 year trends in atrial fibrillation prevalence, incidence, risk factors, and mortality in the Framingham Heart Study: a cohort study. Lancet.

[2] Rahman F, Kwan GF, Benjamin EJ (2014). Global epidemiology of atrial fibrillation. Nat Rev Cardiol.

[3] Grisanti LA (2018). Diabetes and arrhythmias: pathophysiology, mechanisms and therapeutic outcomes. Front Physiol.

[4] Watanabe H, Tanabe N, Watanabe T, Darbar D, Roden DM, Sasaki S, Aizawa Y (2008). Metabolic syndrome and risk of development of atrial fibrillation: the Niigata preventive medicine study. Circulation.

[5] Chan Y-H, Chang G-J, Lai Y-J, Chen W-J, Chang S-H, Hung L-M (2019). Atrial fibrillation and its arrhythmogenesis associated with insulin resistance. Cardiovasc Diabetol.

[6] Wang A, Green JB, Halperin JL, Piccini JP (2019). Atrial fibrillation and diabetes mellitus: JACC review topic of the week. J Am Coll Cardiol.

[7] Seyed Ahmadi S, Svensson AM, Pivodic A, Rosengren A, Lind M (2020). Risk of atrial fibrillation in persons with type 2 diabetes and the excess risk in relation to glycaemic control and renal function: a Swedish cohort study. Cardiovasc Diabetol.

[8] Grant PJ, Cosentino F (2019). The 2019 ESC Guidelines on diabetes, pre-diabetes, and cardiovascular diseases developed in collaboration with the EASD: New features and the ‘Ten Commandments’ of the 2019 Guidelines are discussed by Professor Peter J. Grant and Professor Francesco Cosentino, the Task Force chairmen. Eur Heart J.

[9] Tabák AG, Herder C, Rathmann W, Brunner EJ, Kivimäki M (2012). Prediabetes: a high-risk state for diabetes development. Lancet.

[10] Kim JA, Montagnani M, Koh KK, Quon MJ (2006). Reciprocal relationships between insulin resistance and endothelial dysfunction: molecular and pathophysiological mechanisms. Circulation.

[11] Schlesinger S, Neuenschwander M, Barbaresko J, Lang A, Maalmi H, Rathmann W, Roden M (2022). Prediabetes and risk of mortality, diabetes-related complications and comorbidities: umbrella review of meta-analyses of prospective studies. Diabetologia.

[12] Djupsjö C, Kuhl J, Andersson T, Lundbäck M, Holzmann MJ, Nyström T (2022). Admission glucose as a prognostic marker for all-cause mortality and cardiovascular disease. Cardiovasc Diabetol.

[13] Honigberg MC, Zekavat SM, Pirruccello JP, Natarajan P, Vaduganathan M (2021). Cardiovascular and kidney outcomes across the glycemic spectrum: insights from the UK Biobank. J Am Coll Cardiol.

[14] Jackson AM, Rørth R, Liu J, Kristensen SL, Anand IS, Claggett BL (2022). Diabetes and pre-diabetes in patients with heart failure and preserved ejection fraction. Eur J Heart Fail.

[15] Welsh C, Welsh P, Celis-Morales CA, Mark PB, Mackay D, Ghouri N (2020). Glycated hemoglobin, prediabetes, and the links to cardiovascular disease: data from UK Biobank. Diabetes Care.

[16] Skali H, Shah A, Gupta DK, Cheng S, Claggett B, Liu J (2015). Cardiac structure and function across the glycemic spectrum in elderly men and women free of prevalent heart disease: the Atherosclerosis Risk In the Community study. Circ Heart Fail.

[17] Wang J, Sarnola K, Ruotsalainen S, Moilanen L, Lepistö P, Laakso M (2010). The metabolic syndrome predicts incident congestive heart failure: a 20-year follow-up study of elderly Finns. Atherosclerosis.

[18] Papazoglou AS, Kartas A, Moysidis DV, Tsagkaris C, Papadakos SP, Bekiaridou A (2022). Glycemic control and atrial fibrillation: an intricate relationship, yet under investigation. Cardiovasc Diabetol.

[19] Kezerle L, Tsadok MA, Akriv A, Senderey AB, Bachrach A, Leventer-Roberts M (2021). Pre-diabetes increases stroke risk in patients with nonvalvular atrial fibrillation. J Am Coll Cardiol.

[20] Hsu J-C, Yang Y-Y, Chuang S-L, Chih-Chieh Yu, Lin L-Y (2021). Higher long-term visit-to-visit glycemic variability predicts new-onset atrial fibrillation in patients with diabetes mellitus. Cardiovasc Diabetol.

[21] Hsu J-C, Yang Y-Y, Chuang S-L, Chung Y-W, Wang C-H, Lin L-Y (2021). Underweight is a major risk factor for atrial fibrillation in Asian people with type 2 diabetes mellitus. Cardiovasc Diabetol.

[22] Lee YC, Chao YT, Lin PJ, Yang YY, Yang YC, Chu CC, Wang YC, Chang CH, Chuang SL, Chen WC, Sun HJ (2022). Quality assurance of integrative big data for medical research within a multihospital system. J Formos Med Assoc.

[23] American Diabetes Association Professional Practice Committee (2022). 2 Classification and diagnosis of diabetes: standards of medical care in diabetes-2022. Diabetes Care.

[24] Stekhoven DJ, Bühlmann P (2012). MissForest—non-parametric missing value imputation for mixed-type data. Bioinformatics.

[25] Austin PC (2011). Optimal caliper widths for propensity-score matching when estimating differences in means and differences in proportions in observational studies. Pharm Stat.

[26] Guo C-Y, Yang Y-C, Chen Y-H (2021). The optimal machine learning-based missing data imputation for the cox proportional hazard model. Front Public Health.

[27] Ishwaran H, Kogalur UB, Blackstone EH, Lauer MS (2008). Random survival forests. Ann Appl Stat.

[28] Hsich E, Gorodeski EZ, Blackstone EH, Ishwaran H, Lauer MS (2011). Identifying important risk factors for survival in patient with systolic heart failure using random survival forests. Circ Cardiovasc Qual Outcomes.

[29] American Diabetes Association (2011). Diagnosis and classification of diabetes mellitus. Diabetes Care.

[30] Johansson JS, Boström KB, Hjerpe P, Mourtzinis G, Kahan T, Ljungman C (2022). Prediabetes and incident heart failure in hypertensive patients: Results from the Swedish Primary Care Cardiovascular Database. Nutr Metab Cardiovasc Dis.

[31] Wang Y, O'Neil A, Jiao Y, Wang L, Huang J, Lan Y (2019). Sex differences in the association between diabetes and risk of cardiovascular disease, cancer, and all-cause and cause-specific mortality: a systematic review and meta-analysis of 5,162,654 participants. BMC Med.

[32] Lind V, Hammar N, Lundman P, Friberg L, Talbäck M, Walldius G (2021). Impaired fasting glucose: a risk factor for atrial fibrillation and heart failure. Cardiovasc Diabetol.

[33] Lee SS, Ae Kong K, Kim D, Lim YM, Yang PS, Yi JE (2017). Clinical implication of an impaired fasting glucose and prehypertension related to new onset atrial fibrillation in a healthy Asian population without underlying disease: a nationwide cohort study in Korea. Eur Heart J.

[34] Tabák AG, Brunner EJ, Lindbohm JV, Singh-Manoux A, Shipley MJ, Sattar N (2022). Risk of macrovascular and microvascular disease in diabetes diagnosed using oral glucose tolerance test with and without confirmation by hemoglobin A1c: the Whitehall II Cohort Study. Circulation.

[35] Kim YG, Han KD, Choi JI, Boo KY, Kim DY, Oh SK (2019). The impact of body weight and diabetes on new-onset atrial fibrillation: a nationwide population-based study. Cardiovasc Diabetol.

[36] Karam BS, Chavez-Moreno A, Koh W, Akar JG, Akar FG (2017). Oxidative stress and inflammation as central mediators of atrial fibrillation in obesity and diabetes. Cardiovasc Diabetol.

[37] George AM, Jacob AG, Fogelfeld L (2015). Lean diabetes mellitus: An emerging entity in the era of obesity. World J Diabetes.

[38] Chatterjee R, Kwee LC, Pagidipati N, Koweek LH, Mettu PS, Haddad F (2022). Multi-dimensional characterization of prediabetes in the Project Baseline Health Study. Cardiovasc Diabetol.

[39] Packer M (2020). Do most patients with obesity or type 2 diabetes, and atrial fibrillation, also have undiagnosed heart failure? A critical conceptual framework for understanding mechanisms and improving diagnosis and treatment. Eur J Heart Fail.

[40] Ling LH, Kistler PM, Kalman JM, Schilling RJ, Hunter RJ (2016). Comorbidity of atrial fibrillation and heart failure. Nat Rev Cardiol.

[41] Gopinathannair R, Chen LY, Chung MK, Cornwell WK, Furie KL, Lakkireddy DR (2021). Managing atrial fibrillation in patients with heart failure and reduced ejection fraction: a scientific statement from the American Heart Association. Circ Arrhythm Electrophysiol.

[42] Pickett KL, Suresh K, Campbell KR, Davis S, Juarez-Colunga E (2021). Random survival forests for dynamic predictions of a time-to-event outcome using a longitudinal biomarker. BMC Med Res Methodol.

