# Supplementary material for: Prediabetes as a risk factor for new-onset atrial fibrillation: the propensity-score matching cohort analyzed using the Cox regression model coupled with the random survival forest
Source: Cardiovasc Diabetol. 2023 Feb 20;22:35. doi: 10.1186/s12933-023-01767-x (PMC9940357; doi:10.1186/s12933-023-01767-x)
Supplement: Supplementary file 1 — Additional file 1: Figure S1. propensity score matching with logistic regression (A) and with random forest (B). Figure S2. Concordance between minimal depth and variable of importance (VIMP) for AF. Figure S3. Restrictive cubic spline for hazard ratio between AF and BMI in all patients (3A), model 1 crude for prediabetes (3B), model 2 for prediabetes (3C), model 3 full adjustment for prediabetes (3D). Table S1. Incidence of AF in transition diabetes status (the unmatched cohort). Table S2. C-index between traditional Cox model and random survival forest. Table S3. ICD-code for diagnoses. Table S4. ATC-codes for medications. [file 12933_2023_1767_MOESM1_ESM.docx]

**Supplemental Figure Legends**

Supplemental Figure 1: propensity score matching with logistic regression (A) and with random forest (B)

Supplemental Figure 2: Concordance between minimal depth and variable of importance (VIMP) for AF

Supplemental Figure 3: Restrictive cubic spline for hazard ratio between AF and BMI in all patients (3A), model 1 crude for prediabetes (3B), model 2 for prediabetes (3C), model 3 full adjustment for prediabetes (3D)

Figure S1A


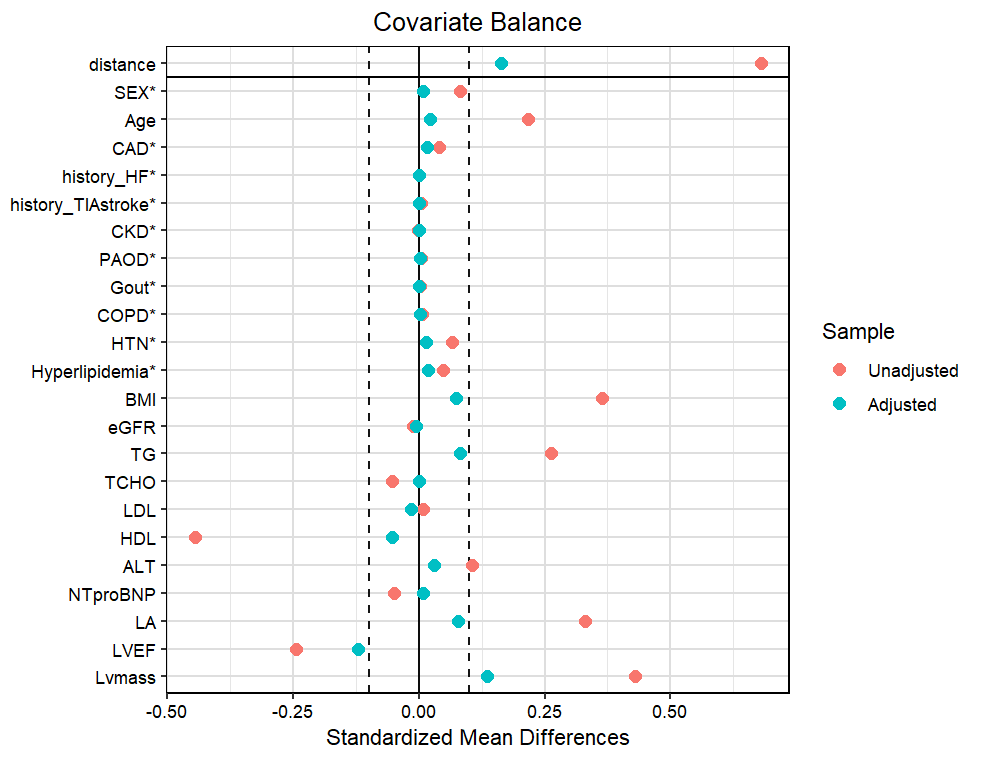


Figure S1B


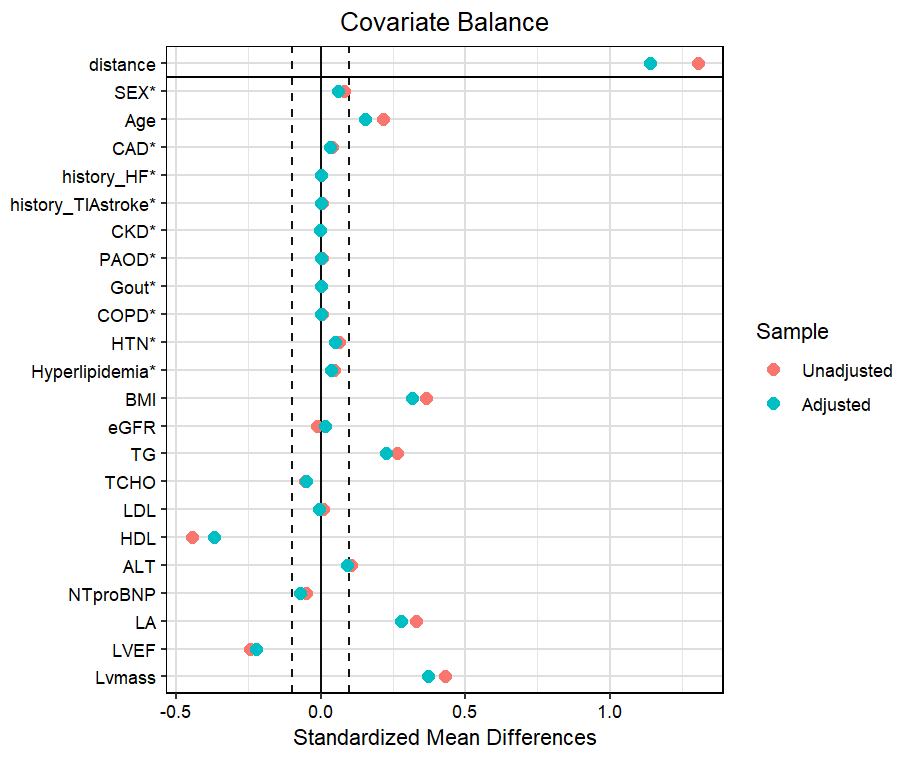


Figure S2


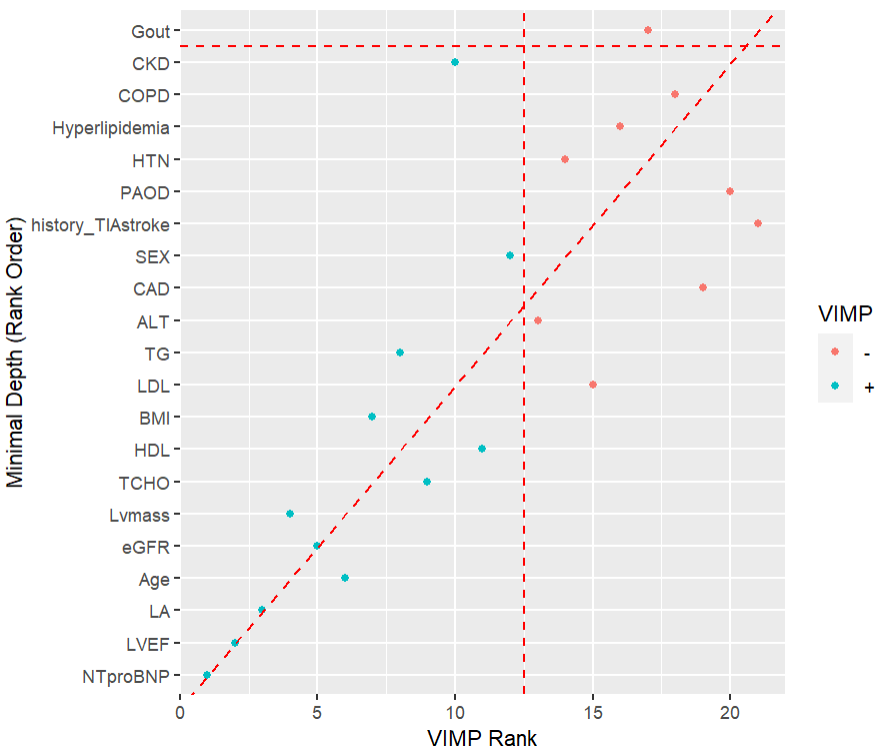


Figure S3A


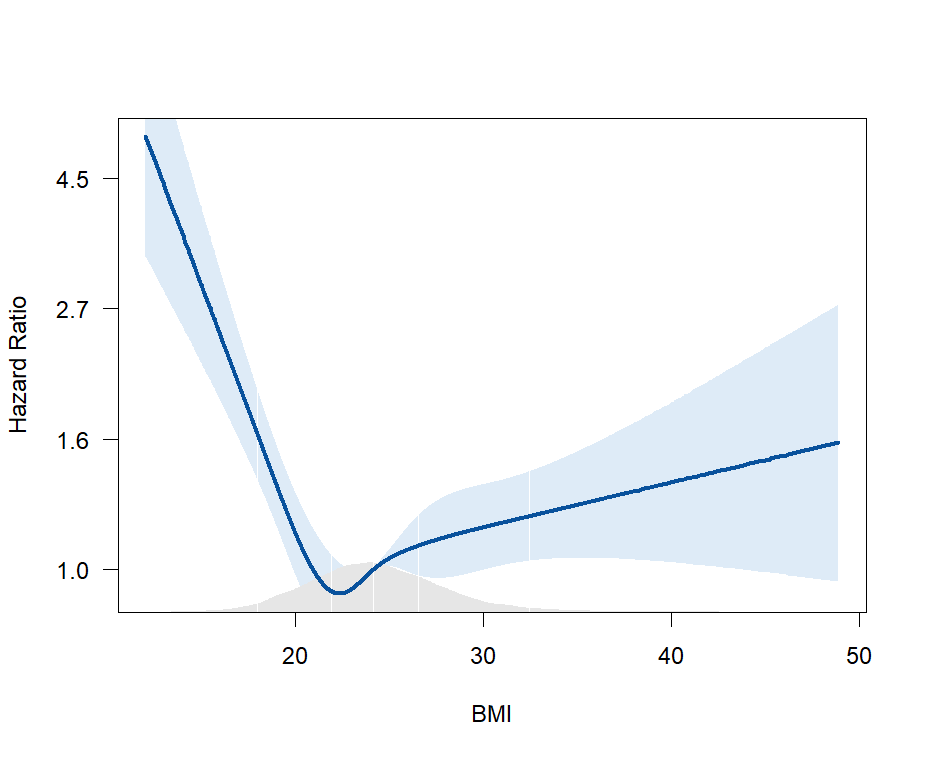


Figure S3B


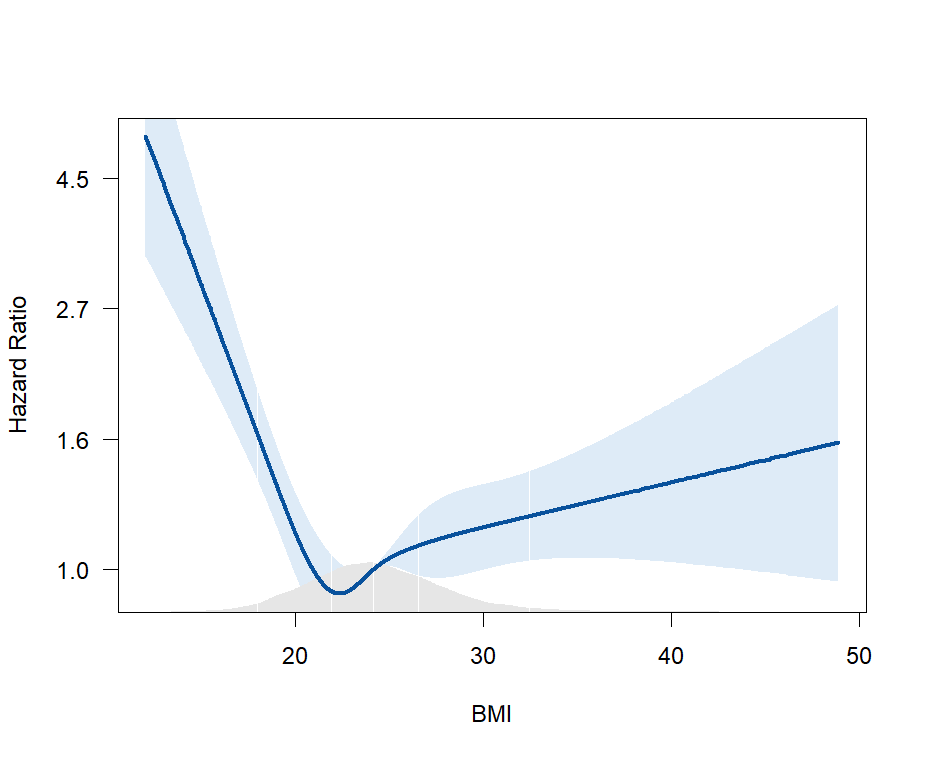


Figure S3C


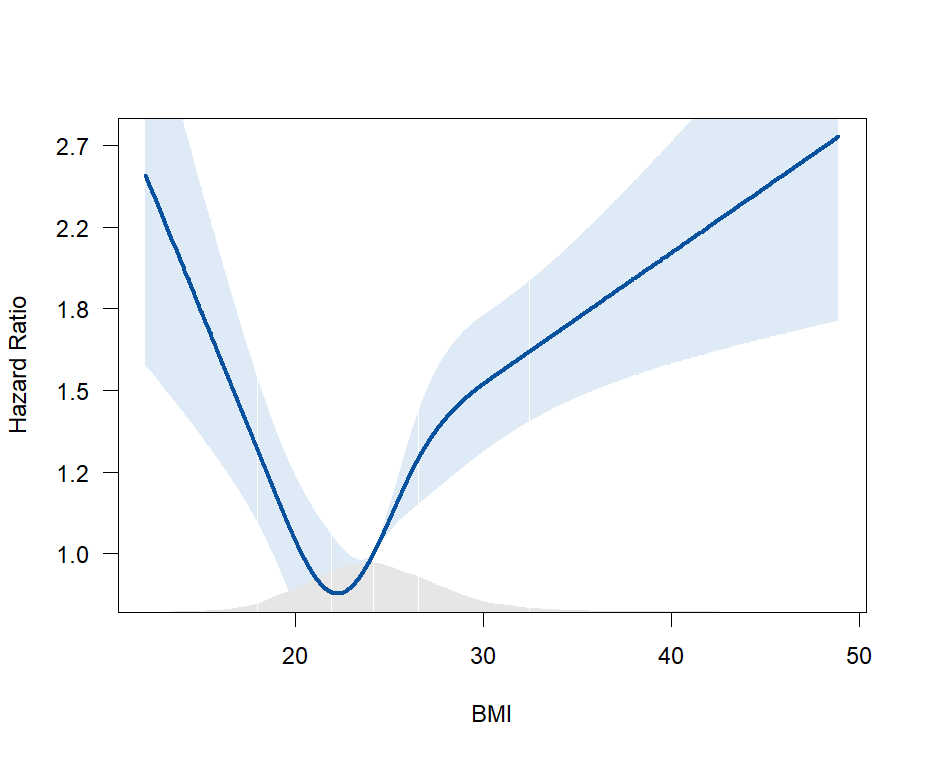


Figure S3D


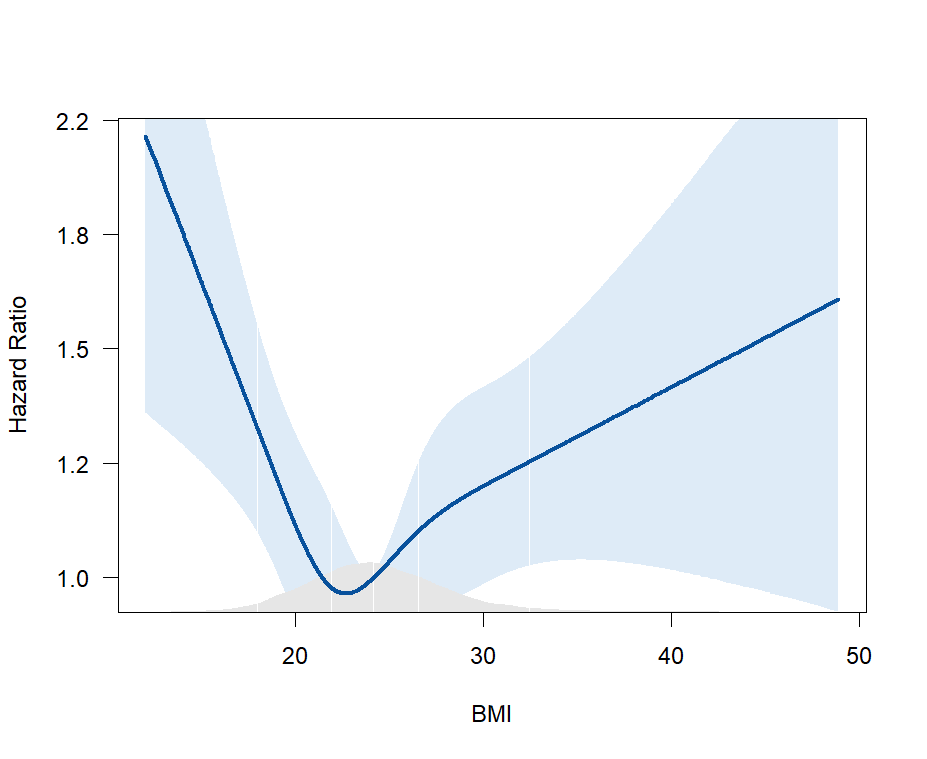


**Table S1.** Incidence of AF in transition diabetes status (the unmatched cohort)

|  | N | Event | Incidence (%) | Age standardized incidence (%) | At risk (person-year) | Median follow-up time (month) | Incidence rate (event/1000 person-year) (95% CI) |
| --- | --- | --- | --- | --- | --- | --- | --- |
| NGT | 18224 | 639 | 3.51 | 2.89 | 64991 | 47.17 | 9.83 (9.10-10.62) |
| With progress | 3603 | 178 | 4.94 | 3.91 | 14924 | 56.70 | 11.92 (10.30-13.80) |
| Without progress | 14621 | 461 | 3.15 | 2.63 | 50067 | 44.37 | 9.21 (8.41-10.08) |
| Prediabetes | 16992 | 798 | 4.70 | 3.32 | 60418 | 46.80 | 13.21 (12.32-14.15) |
| With progress | 2928 | 160 | 5.46 | 3.72 | 12125 | 57.67 | 13.19 (11.31-15.39) |
| Without progress | 14064 | 638 | 4.54 | 3.24 | 43293 | 44.25 | 13.21 (12.23-14.27) |

Abbreviations. NGT, normal glucose test

**Table S2.** C-index between traditional Cox model and random survival forest

| Model for identifying predictors of AF in prediabetes | C-index |
| --- | --- |
| Traditional Cox model | 0.54 |
| Random survival forest | 0.94 |

**Table S3. ICD-code for diagnoses**

ICD-code for diagnoses

|  | ICD-10 code | ICD-9 |
| --- | --- | --- |
| Type 2 diabetes mellitus | E08.XX, E11.XX | 250.XX |
| Atrial fibrillation | I48.0, I48.1, I48.2, I48.91 | 427.31 |
| Atrial flutter | I48.92, I48.4 | 427.32 |
| Hear failure | I50.1. I50.31, I50.9, I50.20, I50.23 | 402.11, 402.91, 404.11, 428.0, 428.1, 428.9 |
| Transient ischemic accident | G45.9, G45.8, G45.4. G45.3 | 435.9 |
| Ischemic stroke | I67.89 | 436 |
| Coronary artery disease | I25.10, I25.11, I25.82, I25.84. I25.9, I20.0, I20.9 | 414.00, 414.01, 414.02, 414.03, 414.04, 414.8, 414.9, 411.81 |
| Chronic kidney disease | E08.22, E11.22, N18.1, N18.2, N18.3, N18.4, N18.5, N18.9, N19 | 593.9 |
| Peripheral arterial occlusive disease | I73.89, I73.9, I74.09, I74.19, I74.2, I74.3, I74.4, I74.9 | 443.81, 443.89, 443.9, 444.0, 444.21, 444.22, 444.81, 444.9 |
| Valvular heart disease | I05.X, I06.X, I07.X, I08.X, I09.X, I34.X, I35.X, I36.X, I37.X | 394.X, 395.X, 396.X, 398.X,397.X, 424.X |
| Gout | M10.00, M10.9, M1A.9X, 274.89 | 274.0, 274.82, 274.11 |
| Chronic obstructive pulmonary disease |  | 491.20, 491.21, 491.8, 492.8. 493.20, 493.22, 493.90. 493.91, 494.0, 494.1, 496 |
| Hypertension | I10, I11.0, I11.9, I12.0, I13.0, I1322 , I15.9 |  |
| Hyperlipidemia | E75.5, E78.0, E78.1, E78.2, E78.3, E78.4, E78.5, E78.9 | 272.0, 272.1, 272.3, 272.4, 272.8 |

**Table S4. ATC-codes for medications**

.

| Medication | ATC codes |
| --- | --- |
| Metformin | A10BA02 |
| Sulphonylurea | A10BB01  A10BB08  A10BB09  A10BB12 |
| Repaglinide | A10BX02 |
| Acarbose | A10BF01 |
| Thiazolidinediones | A10BG03 |
| Sodium-glucose cotransporter-2 inhibitors | A10BD19  A10BH05  A10BK01  A10BK02  A10BK03 |
| Dipeptidyl peptidase-4 inhibitors | A10BH02  A10BH03  A10BH04  A10BH05 |
| Glucagon-like peptide-1 | A10BJ02  A10BJ05 |
| Insulin | A10AB01  A10AB04  A10AB05  A10AC01  A10AD01  A10AD04  A10AD05  A10AE04  A10AE05 |
